# Precision nutrition in MASLD should incorporate metabolic and inflammatory phenotyping

**DOI:** 10.3389/fnut.2026.1820240

**Published:** 2026-06-11

**Authors:** Anastasia Ntikoudi

**Affiliations:** Department of Nursing, University of West Attica, Athens, Greece

**Keywords:** inflammatory, MASLD, metabolic, nutrition, phenotyping

## 1 Introduction

Metabolic dysfunction-associated steatotic liver disease (MASLD) is now recognized as the hepatic manifestation of systemic metabolic dysregulation and represents the most prevalent chronic liver disorder globally. Contemporary clinical practice guidelines emphasize lifestyle modification, particularly dietary intervention and sustained weight reduction, as the cornerstone of management (1, 2). Evidence consistently demonstrates that the magnitude of weight loss correlates with improvement in steatohepatitis features and fibrosis regression in a subset of patients (3). Comprehensive reviews further reinforce that structured dietary modification and physical activity can improve hepatic steatosis and cardiometabolic risk parameters (4, 5). Despite this robust evidence base, clinical response to dietary interventions remains heterogeneous, even among individuals achieving comparable weight reduction.

This variability reflects the biological heterogeneity of MASLD. While insulin resistance is widely recognized as a central pathogenic driver, systemic inflammation, dyslipidemia, adipose tissue dysfunction, and intrahepatic metabolic remodeling contribute in varying degrees to disease progression. Mechanistic studies have demonstrated that liver, muscle, and adipose tissue insulin action is directly related to intrahepatic triglyceride content, highlighting the tight coupling between hepatic fat accumulation and systemic insulin resistance (6, 7). At a molecular level, insulin resistance promotes hepatic *de novo* lipogenesis, increases substrate oversupply, and disrupts lipid partitioning, reinforcing steatosis and metabolic injury (7, 8). In parallel, inflammatory signaling and immune-mediated pathways amplify hepatocellular damage and fibrogenesis (9, 10).

Beyond intrahepatic mechanisms, MASLD reflects a systemic metabolic disorder characterized by complex interactions between insulin resistance, dyslipidemia, adipose tissue dysfunction, and chronic low-grade inflammation (1, 8, 10). These interconnected processes contribute not only to hepatic steatosis and progression to steatohepatitis, but also to broader cardiometabolic risk, including increased cardiovascular disease burden (1, 4). The relative contribution of these mechanisms varies across individuals, giving rise to clinically heterogeneous phenotypes with potentially different therapeutic responses (8, 10). This biological variability provides a strong rationale for moving beyond uniform dietary prescriptions toward phenotype-informed nutritional strategies (4).

Taken together, these interacting metabolic and inflammatory processes generate clinically distinct phenotypic expressions of MASLD, which may reasonably be expected to influence therapeutic responsiveness. However, most dietary trials apply uniform nutritional regimens without stratifying participants according to baseline metabolic or inflammatory burden. Such an approach may dilute signals in responsive subgroups and limit progress toward precision nutrition.

This Opinion argues that metabolic and inflammatory phenotyping, grounded in accessible clinical biomarkers such as insulin resistance indices, triglyceride-to-HDL cholesterol (TG/HDL-C) ratio, and high-sensitivity C-reactive protein (hs-CRP), should inform dietary strategy selection and future MASLD trial design.

## 2 Insulin resistance as a heterogeneous driver of MASLD

Insulin resistance is a defining feature of MASLD pathophysiology. Early metabolic investigations demonstrated that insulin action in liver, skeletal muscle, and adipose tissue declines proportionally with increasing intrahepatic triglyceride content, underscoring the systemic nature of hepatic steatosis (6). At a molecular level, impaired insulin signaling enhances hepatic *de novo* lipogenesis through activation of lipogenic transcription factors while reducing suppression of adipose tissue lipolysis, thereby increasing free fatty acid flux to the liver (8, 10). This metabolic environment promotes triglyceride accumulation within hepatocytes and contributes to progression toward steatohepatitis.

More recent studies provide direct evidence that insulin resistance drives hepatic *de novo* lipogenesis in MASLD. Korenblat et al. (6) and Smith et al. (7) demonstrated that hepatic *de novo* lipogenesis is markedly increased in insulin-resistant individuals and strongly correlated with intrahepatic triglyceride content. These findings support the concept that hyperinsulinaemia and hyperglycaemia are not merely associated features but active drivers of hepatic lipid synthesis. Importantly, the contribution of *de novo* lipogenesis to hepatic fat appears particularly pronounced in metabolically dysregulated states.

Dietary macronutrient composition directly influences these pathways. Excessive intake of simple carbohydrates, particularly fructose, stimulates *de novo* lipogenesis, whereas saturated fat intake contributes to lipotoxicity and mitochondrial dysfunction (11). A comprehensive review of dietary carbohydrates and fats in NAFLD emphasized that macronutrient quality and metabolic context critically shape hepatic lipid handling (11). However, MASLD is not synonymous with uniform insulin resistance severity. Comparative analyses of surrogate indices, including HOMA-IR, the triglyceride-glucose index, and TG/HDL-C ratio, demonstrated heterogeneity in their discriminative performance across populations (12, 13). Some individuals exhibit profound insulin resistance and atherogenic dyslipidemia, whereas others present with hepatic steatosis despite comparatively modest systemic metabolic impairment.

This heterogeneity has direct implications for nutritional strategy. Interventions primarily targeting glycemic load and insulin sensitivity may yield greater benefit in metabolically dominant phenotypes than in individuals whose disease trajectory is more strongly influenced by inflammatory or genetic mechanisms. Routine reporting of baseline insulin resistance metrics, or validated lipid-based surrogates such as TG/HDL-C ratio, would enhance interpretability of dietary trials and facilitate phenotype-stratified analyses.

## 3 Systemic inflammation as a critical stratification axis

Chronic low-grade inflammation is increasingly recognized as a pivotal contributor to MASLD progression. Adipose tissue macrophage infiltration, dysregulated adipokine secretion, and cytokine-mediated signaling promote hepatocellular injury and fibrogenesis (9, 10). The pathogenesis of MASLD has been comprehensively reviewed as a convergence of metabolic overload and inflammatory activation, in which lipotoxic intermediates, oxidative stress, and immune signaling drive progression from steatosis to steatohepatitis (10).

High-sensitivity CRP, although not liver-specific, provides a widely available measure of systemic inflammatory tone. A recent population-based, cross-sectional analysis across steatotic liver disease phenotypes demonstrated variation in hs-CRP levels, supporting its relevance for clinical characterization (14). Associations between CRP levels, histological, and elastographic indices of NAFLD in individuals with severe obesity further reinforce the role of inflammatory burden in disease severity (15). Importantly, inflammatory markers do not consistently parallel insulin resistance severity, suggesting partially distinct mechanistic trajectories.

Dietary modulation of inflammation is biologically plausible. Mediterranean-style dietary patterns rich in fiber, polyphenols, and unsaturated fats have demonstrated favorable effects in NAFLD populations (16). Moreover, a systematic evaluation of randomized controlled trials comparing low-fat and low-carbohydrate calorie-restricted diets indicates that while weight reduction remains the principal driver of improvement in liver enzymes and hepatic fat content, no clear consensus exists regarding optimal macronutrient composition, underscoring persistent uncertainty in dietary prescription (17). Given the established links between diet composition, insulin signaling, and inflammatory pathways (11), incorporation of inflammatory stratification into dietary trials represents a logical extension of precision nutrition principles.

## 4 Discussion

A pragmatic approach to precision nutrition in MASLD may be operationalized through a two-axis stratification model incorporating insulin resistance and systemic inflammatory burden. Such an approach reflects the biological heterogeneity of MASLD and provides a clinically accessible framework to support more targeted dietary strategies.

Insulin resistance may be approximated using surrogate indices such as lipid-derived markers, including the triglyceride-to-HDL cholesterol (TG/HDL-C) ratio, which has been associated with insulin resistance and cardiometabolic risk across diverse populations (18–20). Where feasible, the homeostasis model assessment of insulin resistance (HOMA-IR) may provide additional insight, although its clinical utility is limited by the requirement for fasting insulin measurement and the absence of universally accepted thresholds (21, 22). In parallel, systemic inflammatory burden may be approximated using high-sensitivity C-reactive protein (hs-CRP), with established cardiovascular risk categories (< 1 mg/L, 1–3 mg/L, >3 mg/L) offering a pragmatic framework for stratification in clinically stable individuals (23). Although non-specific, hs-CRP reflects low-grade inflammation that is central to the pathophysiology of steatotic liver disease and its cardiometabolic complications (24).

Integrating these axes yields a pragmatic four-phenotype framework comprising metabolic-dominant, inflammatory-dominant, mixed, and low-burden profiles. This classification is not intended as a rigid taxonomy, but rather as a tool to contextualize inter-individual variability in disease expression and therapeutic responsiveness. This framework is intended as a hypothesis-generating clinical tool rather than a definitive classification system. From a practical perspective, these phenotypes may inform dietary emphasis. Within this framework, dietary strategies may be qualitatively tailored. Individuals with metabolically dominant profiles may derive greater benefit from interventions targeting glycaemic load and insulin sensitivity, whereas inflammatory-dominant phenotypes may respond preferentially to dietary patterns with established anti-inflammatory properties, such as Mediterranean-style diets (4, 16, 25). Mixed phenotypes may require combined strategies that address both metabolic and inflammatory drivers.

These phenotype-informed approaches should complement, rather than replace, established lifestyle recommendations. The magnitude of weight loss remains the principal determinant of histological improvement in MASLD, with consistent evidence demonstrating that greater weight reduction is associated with higher likelihood of steatohepatitis resolution and fibrosis improvement (1, 3). Accordingly, stratification should be viewed as a means of refining dietary composition within a shared therapeutic foundation centered on weight reduction.

From a research perspective, incorporation of baseline metabolic and inflammatory phenotyping into dietary intervention trials may enhance interpretability and reduce apparent heterogeneity in outcomes. Standardized reporting of insulin resistance markers, lipid ratios, and hs-CRP would enable pre-specified subgroup analyses and facilitate exploration of effect modification. Such an approach aligns MASLD nutritional research with broader precision medicine paradigms and may improve identification of phenotype-specific responses to dietary interventions.

A further dimension reinforcing the systemic nature of MASLD is its applicable association with subclinical vascular disease. Accumulating evidence indicates that hepatic steatosis is linked to early atherosclerotic changes through shared mechanisms including insulin resistance, chronic inflammation, oxidative stress, and atherogenic dyslipidemia (26–29). Carotid intima-media thickness (cIMT) is a widely used, non-invasive surrogate marker of subclinical atherosclerosis and cardiovascular risk, and has been shown to be increased in individuals with hepatic steatosis (30). While several studies suggest associations between hepatic steatosis severity, inflammatory mediators, and vascular remodeling, the strength and independence of these relationships vary across populations and study designs (31–34). From a clinical perspective, dietary interventions that improve insulin sensitivity and reduce hepatic fat content may concurrently attenuate systemic inflammation and vascular risk, extending the impact of nutritional strategies beyond the liver.

## 5 Conclusion

Overall, MASLD represents a biologically heterogeneous condition characterized by variable contributions of insulin resistance and systemic inflammation, mechanisms that are unlikely to respond uniformly to identical dietary prescriptions. Continued reliance on non-stratified nutritional strategies risks obscuring phenotype-dependent responsiveness and limiting therapeutic precision. A pragmatic metabolic-inflammatory phenotyping framework grounded in accessible clinical biomarkers offers a feasible pathway toward operationalizing precision nutrition in MASLD. Prospective validation within stratified clinical trials should therefore be considered a methodological priority to determine whether such an approach enhances therapeutic alignment and improves the interpretability of dietary intervention studies.
